# Beyond the Scale: Investigating Adiponectin, ICAM-1, and VCAM-1 as Metabolic Markers in Obese Adolescents with Metabolic Syndrome

**DOI:** 10.1155/2023/4574042

**Published:** 2023-10-03

**Authors:** Nur Aisiyah Widjaja, Leonardo Alexander Caesar, Suhasta Nova, Eva Ardianah

**Affiliations:** ^1^Faculty of Medicine, Child Health Department, Universitas Airlangga, Jl. Mayjen Prof. Dr. Moestopo No. 47, Surabaya 60132, Indonesia; ^2^Ikatan Dokter Indonesia Surabaya, Jl. Mayjen Prof. Dr. Moestopo No. 117, Surabaya 60132, Indonesia

## Abstract

**Background:**

Adiponectin acts to prevent vascular dysfunction due to obesity by inhibiting ICAM-1 and VCAM-1 expressions.

**Objective:**

We investigate adiponectin ICAM-1, VCAM-1, and metabolic syndrome (MetS) in obese adolescents.

**Methods:**

A cross-sectional study with healthy obese adolescents aged 13 to 18 years was conducted from October 2019 to January 2020. Statistical analysis conducted was a test of normality and homogeneity tests, ANOVA/Kruskal–Wallis, independent sample *T*-test/Mann–Whitney *U* test, and Spearman correlation and determined as significant if *p* value <0.05.

**Results:**

125 obese adolescents were recruited. 42 (33.6%) were obese with MetS (we grouped as MetS) and 83 (66.4%) subjects without MetS (non-MetS group). VCAM-1 was significantly higher on boys with MetS compared to girls with MetS, and even girls with MetS had lower levels of VCAM-1 than boys with non-MetS. ICAM-1 was significantly higher in boys with low-level HDL-c (*p* < 0.05) and correlated weakly with HDL-c, while adiponectin levels were significantly lower in girls with central obesity and hypertriglyceridemia. Path analysis showed that triglyceride had a direct effect on ICAM-1 but not VCAM-1 in both obese boys and girls. Adiponectin had a negative direct effect on ICAM-1 and VCAM-1 in girls. However, on boys, diastole blood pressure had a negative direct effect, which might be the role of sex hormones indirectly.

**Conclusion:**

VCAM-1 was significantly higher in boys than girls, which showed that boys had a higher risk of atherosclerosis. ICAM-1 showed no significant difference in both gender and metabolic states. Adiponectin showed a protective effect by lowering ICAM-1 and VCAM-1 directly on girls.

## 1. Introduction

Adipose tissues secreting a number of adipokines regulate insulin sensitivity, energy metabolism, and vascular homeostasis, so the dysfunction of the adipose tissue is linked with the incidence of obesity accompanied with insulin resistance, hypertension, and cardiovascular disease [1]. Obesity is known to alter the expression of adipokines due to the adipose tissue hypertrophy, including adiponectin, in which it is able to exert a potent anti-inflammatory and vascular protective effect [2]. Adiponectin is one of the most abundant adipokines known as the obesity hormone. and the levels of adiponectin in the circulation was 3 *μ*g/mL to 30 *μ*g/mL in humans [3]. In a study, adiponectin is associated with body weight and abdominal obesity and MetS risk factor, especially low-level HDL-c and hypertriglyceridemia [4].

It has been proposed that adiponectin acts to prevent the vascular dysfunction due to obesity and diabetes by improving insulin sensitivity and metabolic profiles to reduce the risk factors for cardiovascular disease and protects the vasculature through its pleiotropic actions on endothelial cells, endothelial progenitor cells, smooth muscle cells, and macrophages [1]. The concentrations of adiponectin of 5 to 25 mg/mL had a significant inhibitory effect on the expression of monocyte adhesion and adhesion molecule induced by TNF-*α* in vitro. Atherosclerosis is an inflammatory disease in which adhesion molecules on arterial endothelial cells are responsible for the accumulation of monocytes/macrophages and T lymphocytes. Obesity is a low-grade inflammation which causes endothelial dysfunction process, by increasing free radicals derived from oxygen. The free radicals was produced by adipocyte hypertrophy. The imbalance of ROS and antioxidant system causes an oxidative stress which leads to endoplasmic reticulum (ER) stress and mitochondrial dysfunction [5]. Adiponectin is accumulated in the vasculature, and it reduced on obesity due to suppression by TNF-*α* and lead to adiponectin-deficiency, which stimulate the significant increases of vascular cell adhesion molecule 1 (VCAM-1) and intercellular adhesion molecule 1 (ICAM-1) or known as CD54 in aortic intima [6].

Here, we investigate the levels of adiponectin, ICAM-1, and VCAM-1 with the incidence of MetS in obese adolescents.

## 2. Materials and Methods

### 2.1. Ethical Approval

The study has been approved and declared as ethically appropriate by the Research and Ethics Scientific Committee 23/EC/KEPK/FKUA/2021 released by Faculty of Medicine, Airlangga University, Surabaya, Indonesia. Screening of obesity was performed with the permission of the head school and parents of the subjects.

### 2.2. Study Design

A cross-sectional study with healthy obese adolescents aged 13–18 years was conducted from October 2019 to January 2020. Obesity was determined based on the body mass index (BMI) for age based on gender ≥percentile 95th of CDC growth chart 2000.

### 2.3. Samples

The subjects must be healthy (did not take corticosteroids 6 months before the study or dyslipidemia medication 3 months before the study, did not take antibiotic or hormonal therapy, did not smoke or consume alcohol, and did not have autoimmune disease or endocrinology disorders).

Sample size was measured with

(1) $$\begin{array}{c} n=\frac{Z_{1-\alpha /5}^{2}P\left(1-P\right)}{d^{2}}, \end{array}$$

where *n* is the sample number, *P* is the subject proportion in the study = 0.5, *d*^2^ is the absolute value = 0.1, *Z*_1−*α*/5_ is the expected significant = 1.96, CI is the confidence interval = 95%, and *n* = 97 subjects.

### 2.4. Blood Sample Examination

Blood samples were withdrawn via cubital vein as much as 5 ml by the trained analyst and placed on vacutainer with EDTA. After that, the vacutainer was replaced in an icebox to transport to the laboratory. Blood analysis includes lipid profile, fasting blood glucose, and fasting insulin by a designated medical laboratory. For adiponectin, ICAM-1, and VCAM-1 analyses, the blood that has been taken was not immediately analysed. The blood was centrifuged to remove the serum and then stored at −70°C until the analysis was performed in the laboratory. Adiponectin was analysed using the Human Adiponectin ELISA kit (Bioassay Technology Laboratory). ICAM-1 was analysed using the Human Intercellular Adhesion Molecule 1 ELISA kit (Bioassay Technology Laboratory) while sVCAM-1 was analysed using the Human Vascular Cell Adhesion Molecule-1 ELISA kit (Bioassay Technology Laboratory).

### 2.5. Anthropometry Measurements

Body weight was measured using Seca Robusta 813 digital scale in standing position, by stepping on the scale w,hile the body height was measured using Seca 206 Body Meter. Height was measured from the vertex of the head to the heel in standing position. Waist circumference and hip circumference were measured using Seca 201 measuring tape. Waist circumference was measured by wrapping the measuring tape around the subject's stomach, at the midpoint between the lowest rib and the endpoint of the iliac crest upon expiration, in line with the navel. Hip circumference was measured by asking the subjects to keep the feet together, then wrapping the measuring tape around the widest part of hips, at the point of the greatest gluteal protuberance. The subjects were measured using light cloth without footwear and other accessories such as belt, hat, or hair accessories. BMIs were calculated by dividing body weight (in kg) with body height square (in m) (body weight (kg)/body height^2^ (m^2^)), in kg/m^2^ unit.

Blood pressure was measured using Omron Automatic Blood Pressure Monitor HEM-8712 (Omron Health Care Co., Ltd, Japan) by placing the cuff on the right arm and then pulling and tightening it according to the size of the arm. After it was installed correctly (fastened and did not move), the power button on the digital tension tool was pressed, and then the microprocessor started to drive air pressure into the cuff, and then the value of blood pressures will appear in the manometer tube column. Blood pressure measurement was performed in a sitting position after the subject had rested for 10 minutes.

### 2.6. Determination of MetS on the Subject

Metabolic syndrome criteria were determined using the International Diabetic Federation criteria [7] and central obesity (defined as waist circumference ≥88 cm for boys and ≥85 cm for girls aged 10–16 years, and ≥94 cm for boys and ≥80 cm for girls aged >16 years) accompanied by at least two or more of the following: hypertension (defined as systole blood pressure ≥ 130/diastole blood pressure ≥ 85 mmHg), hypertriglyceridemia (defined as triglyceride levels ≥110 mg/dl for adolescents aged 10–16 years and ≥150 mg/dl for adolescents >16 years), low-level HDL-c (defined as HDL-c levels ≤40 mg/dl, <50 mg/dl for girls aged 10–16 years, <40 mg/dl for boys, and <50 mg/dl for girls aged >16 years), and hyperglycaemia (defined as fasting blood glucose (FBG) levels ≥110 mg/dl for adolescents aged 10–16 years and was ≥100 mg/dl for adolescents aged >16 years) [8].

### *2.7*. Statistical Analysis

Statistical analyses conducted were test of normality and homogeneity test, ANOVA/Kruskal–Wallis, independent sample *T*-test/Mann–Whitney *U* test, and Spearman correlation and determined as significant if *p* value <0.05.

## 3. Results

A total of 125 obese adolescents were recruited to follow this study, 88 were boys and 37 were girls. 42 (33.6%) of the subjects were obese with metabolic syndrome (MetS), so we grouped as the MetS group, while 83 (66.4%) did not had MetS, and we grouped as the non-MetS group. The incidence of MetS on boys was 29.6%, while on girls only 4%. The age of the subjects was almost similar in both groups ranging from 147 to 220 months in MetS and 152 to 224 months in non-MetS. The gender distribution showed that boys were predominant on MetS compared to non-MetS (83.78% vs. 61.45%; *p*=0.019). The characteristics of subjects on both groups are summarized in Table 1.

There was a significant difference on body weight (*p*=0.003). Boys with MetS were significantly heavier than boys with non-MetS (92.64 ± 14.43 vs. 84.82 ± 13.51 kg; *p*=0.032) and girls with non-MetS (92.64 ± 14.43 vs. 78.30 ± 9.44 kg; *p* ≤ 0.001). There was a significant difference on body height (*p* ≤ 0.001), boys with MetS were significantly taller than boys with non-MetS (167.38 ± 8.56 vs. 162.76 ± 7.38 cm; *p*=0.025) and girls with non-Mets (167.38 ± 8.56 vs. 156.05 ± 5.71 cm; *p* ≤ 0.001). There was a significant difference on waist circumference (*p* ≤ 0.001), boys with MetS had significantly larger waist circumference than girls with MetS (103.33 ± 7.91 vs. 91.50 ± 5.15 cm; *p*=0.010) and girls with non-MetS (103.33 ± 7.91 vs. 90.13 ± 8.73 cm, *p* ≤ 0.001). And boys with non-MetS had significantly larger waist circumference than girls with non-MetS (99.92 ± 9.17 vs. 90.13 ± 8.73 cm; *p* ≤ 0.001).

There was a significant difference on systole blood pressure on girls with MetS compared to girls with non-MetS (132.00 ± 8.37 vs. 118.75 ± 9.07 mmHg; *p*=0.009). While on boys with MetS, the systole blood pressure was significantly higher than boys with non-MetS (130.54 ± 10.46 vs. 120.39 ± 13.55 mmHg; *p* ≤ 0.001) and girls with non-MetS (130.54 ± 10.46 vs. 118.75 ± 9.07 mmHg; *p* ≤ 0.001). On diastole blood pressure, there was a significant difference on girls with MetS compared to girls with non-MetS (86.00 ± 8.94 vs. 77.18 ± 6.83 mmHg; *p*=0.024). Boys with MetS had significantly higher diastole blood pressure than boys with non-MetS (86.35 ± 10.32 vs. 77.55 ± 8.74 mmHg; *p* ≤ 0.001) and girls with non-MetS (86.35 ± 10.32 vs. 77.18 ± 6.83 mmHg; *p* ≤ 0.001).

There was a significant difference on HDL-c levels (*p* ≤ 0.001), which was lower on boys with MetS than boys with non-MetS (37.30 ± 5.41 vs. 44.47 ± 6.29 mg/dL; *p* ≤ 0.001) and girls with non-MetS (37.30 ± 5.41 vs. 46.03 ± 7.62 mg/dL; *p* ≤ 0.001). The triglycerides level was higher on boys with MetS than boys with non-MetS (148.10 ± 68.57 vs. 102.72 ± 62.60 mg/dL; *p* ≤ 0.001) and girls with non-MetS (148.10 ± 68.57 vs. 101.81 ± 63.24 mg/dL; *p*=0.005). Girls with MetS had significantly higher levels of triglycerides than boys with non-MetS (149.60 ± 36.39 vs. 102.72 ± 62.60 mg/dL; *p*=0.026) and girls with non-MetS (149.60 ± 36.39 vs. 101.81 ± 63.24 mg/dL; *p*=0.023).

There was a significant difference on fasting insulin (*p*=0.021), which was higher on boys with MetS than boys with non-MetS (28.91 ± 18.53 vs. 18.74 ± 7.57 mU/L; *p*=0.001) and girls with non-MetS (28.91 ± 18.53 vs. 21.13 ± 11.45 mU/L; *p*=0.021). The value of HOMA IR on boys with MetS was significantly higher than boys with non-MetS (6.20 ± 4.02 vs. 3.94 ± 1.58; *p*=0.003) and girls with non-MetS (6.20 ± 4.02 vs. 4.45 ± 2.35; *p*=0.049).

The VCAM-1 levels were higher on boys with MetS than girls with MetS (15.57 ± 22.24 vs. 4.52 ± 1.45 pg/mL; *p*=0.003) and girls with non-MetS (15.57 ± 22.24 vs. 5.28 ± 6.99 pg/ml; *p* ≤ 0.001) while girls with MetS had a significantly lower levels of VCAM-1 than boys with non-MetS (4.52 ± 1.45 vs. 13.41 ± 13.04 pg/ml; *p* ≤ 0.001). Girls with non-MetS had significantly lower levels of VCAM-1 than boys with non-MetS (5.28 ± 6.99 vs. 13.41 ± 13.04 pg/ml; *p* ≤ 0.001).

Table 2 summarizes the levels of adiponectin, ICAM-1, and VCAM-1 on the subjects based on MetS risk factors on boys. There was no significant difference on adiponectin, ICAM-1, and VCAM-1 between the subjects with central obesity and hyperglycemic compared to noncentral obesity and nonhyperglycemic (*p* > 0.05). There was a significant difference of ICAM-1 levels on low-level HDL-c subjects compared to normal HLD-c (3,138.68 ± 15,018.66 vs. 995.04 ± 1,567.86 pg/ml; *p* < 0.05). There was a significant difference of VCAM-1 levels on the boys with hypertension, lower on the hypertension boys compared to nonhypertension boys (9.01 ± 4.98 vs. 18.17 ± 21.78 pg/ml; *p* ≤ 0.001).

Table 3 summarizes the level of adiponectin, ICAM-1, and VCAM-1 on the subjects based on MetS risk factors on girls. There was no significant difference of adiponectin, ICAM-1, and VCAM-1 on the subject with hypertension, hyperglycaemia, and low level of HDL-c (*p* < 0.005). On girls with central obesity, the adiponectin levels were significantly lower than noncentral obesity (14.25 ± 6.09 vs. 22.53 ± 13.36 ng/ml; *p*=0.016) and on girls with hypertriglyceridemia (13.93 ± 9.91 vs. 16.83 ± 7.45 ng/ml; *p*=0.042).

Table 4 summarizes the correlation of MetS parameters with ICAM-1, VCAM-1, and adiponectin based on the gender. On boys, there was a negative very week correlation between adiponectin and fasting insulin (*r* = −0.272; *p*=0.011), while ICAM-1 had a very weak correlation with fasting blood glucose (*r* = 0.217; *p*=0.043) and HDL-c levels (*r* = −0.261; *p*=0.016). VCAM-1 levels were correlated negatively with systolic (*r* = −0.265; *p*=0.013) and diastolic blood pressure (*r* = −0.264; *p*=0.013) and had very weak positive correlation with LDL-c levels (*r* = 0.228; *p*=0.033). While on girls, there was a weak correlation between ICAM-1 with fasting blood glucose (*r* = 0.422; *p*=0.009).

**Figure 1:**
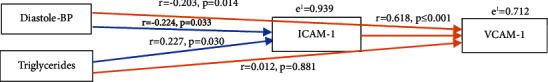
Path analysis of the effect of diastole-BP and triglycerides on ICAM-1 and VCAM-1 in boys.

Path analysis to analyse the effect of MetS components (waist circumference, fasting blood glucose, triglycerides, HDL-c, and systole and diastole blood pressure) on ICAM-1 showed that diastole-BP and triglyceride levels had a direct effect on ICAM-1 in boys, as summarized in Figure 1, while insulin and adiponectin levels did not show their effect on ICAM-1 or VCAM-1 directly or indirectly.

Diastole-BP and triglyceride levels had a significant direct effect on ICAM-1 (*p* < 0.05). Diastole-BP also had a significant direct effect on VCAM levels, but triglycerides did not exert the same effect (*p* > 0.05). Both MetS components, diastole-BP and triglycerides, did not have a indirect effect on VCAM-1 via ICAM-1 (the value of indirect effect < direct effect).

**Figure 2:**
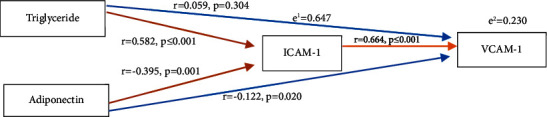
The effect of triglycerides and adiponectin levels to VCAM-1 and VCAM-1 in girls.

On girls, the path analysis of MetS variables is summarized in Figure 2, which showed that triglyceride and adiponectin levels exert its effect on ICAM-1 directly (*p* < 0.05). Adiponectin had a negative direct effect on VCAM-1 (*p* < 0.05), but triglyceride did not exert its direct effect on VCAM-1 (*p* > 0.05). Both triglycerides and adiponectin did not have an indirect effect on VCAM-1 via ICAM-1 (the value of indirect effect < direct effect).

## 4. Discussion

Obesity and metabolic syndrome in children and adolescents are associated with adverse consequences in adulthood, especially coronary heart disease due to overproduction of inflammatory mediators and insulin resistance [9]. The prevalence of MetS was 4.8% in boys and 3.4% in girls according to the study conducted in South Korea, higher in girls aged more than 12 years; however, on boys, higher in those aged 13 years or above [10], which was lower than this study, especially in boys. The prevalence of MetS was increased 17% in boys during nine years (2010–2019) in Arab adolescents, higher than in girls, which count only 2.9%, and the prevalence was higher in boys [11], which is similar with this study. Other study conducted in Tehranian children and adolescents showed the same results; the prevalence on boys was increased by 3.2% during phase I (1999–2001) to phase III (2006–2008), while on girls, the prevalence was decreased by 5.2% [9]. However, the changes on dietary practices and sedentary lifestyle were suspected to increase the obesity prevalence in developing countries [12]. Factors influenced the high prevalence in boys was the increasing of age, meal habits (having one meal per day), and body awareness (weight maintenance) [11].

Excess body weight leads to overweight and obesity [13], which contributes to the risk factor of MetS in early onset, on adolescents [14, 15]. The prevalence of MetS in obese adolescents was 12.4% to 44.2%, and in some populations, it reaches 50% [16], which was in line with this study. The incidence of MetS was developed due to the interaction between obesity, insulin resistance, and inflammation [17]. The BMI could estimate the body fat indirectly and an excellent screening method to predict obesity on paediatric population, but it cannot be used as single diagnostic tools [18].

Fasting insulin levels are higher in MetS. Gender, puberty stage, BMI, and waist circumference have a correlation with fasting insulin levels [19]. In healthy conditions, glucose is transported into myosin by GLUT-4 under the influence of insulin through the process of Glut4 translocation [20] which is then phosphorylated into glucose-6-phosphate. This intermediate product is then stored as glycogen or becomes a substrate in the glycolytic pathway [21]. In obesity, fat tissues, especially in the abdominal area, are prone to lipolysis which causes an increase in free fatty acids (FFA) in the body. High concentrations of FFA mediated IR by reducing the strength of insulin signals through insulin receptor substrate (IRS)-1/phosphatidylinositol (PI) 3-kinase pathway [22]. In addition, adipokines produced by adipocytes such as resistin and retinol-binding protein 4 which reduced the insulin sensitivity [23].

The pathogenesis of endothelium inflammation was triggered by cytokines in which the endothelium is stimulated, so that the endothelium is more adhesive for leucocytes and leads to the development of inflammatory lesions. The leucocyte adhesion to the vascular endothelium is the most important process of the leucocyte extravasation during the inflammation process in which it is mediated by E-selectin. The unity and transendothelial migration depend on the interaction between adhesion molecule-1 (ICAM-1), vascular cell adhesion molecule-1 (VCAM-1), and the ligands [24]. ICAM-1 and VCAM-1 have functions as the adhesion of leucocyte to the vascular endothelium [25], by facilitating the leukocyte transmigration across the endothelium [26]. So, both ICAM-1 and VCAM-1 have been used as inflammation marker and endothelial dysfunction [27] because the expression of both CAMs is enhanced by a variety of proatherogenic stimuli such as proinflammatory cytokines and reactive oxygen species [28]. Obesity induces early endothelial activation [29] due to its low-grade chronic inflammation [29] and increases the expression of adhesion molecules mRNA and protein levels in the visceral adipose tissue, which is linked to central obesity (visceral adiposity) and the risk of cardiovascular diseases [30].

However, the study of CAMs on paediatric population is still in controversial results. A study in Vienna, Austria, showed that both ICAM-1 and VCAM-1 showed no significant difference in lean or obese children [31]. On healthy obese adult, it has been proved that sICAM-1, E-selectin, and P-selectin were significantly higher than those in healthy lean adults, but there is no significant difference on sVCAM-1 serum concentrations on both groups [32]. While in this study, there is no significant difference between MetS and non-MetS groups due to the subject being healthy obese adolescents. Other study investigating sVCAM-1 concentration on healthy subjects with myocardial infarction supported this finding; it showed there is no significant difference between control and case subjects, which are similar with this study [33].

Intercellular adhesion molecule-1 (ICAM-1) and vascular cell adhesion molecule-1 (VCAM-1) are transmembrane immunoglobulins in which they are upregulated during the endothelial activation. Both ICAM-1 and VCAM-1 have adhesion molecules on leucocytes (monocytes, lymphocytes, and neutrophils) as ligand that are CD11⁄CD18 for ICAM-1 and very late antigen-4 (VLA-4) for VCAM-1 [34], in which they play a critical role in monocyte adherence to endothelial cells. ICAM-1 and VCAM-1 are overexpressed on endothelial lumen in many pathological states, and the level is increased in atherosclerotic lesion [25]. The average of soluble ICAM-1 was significantly higher in individuals with low-level of HLD-c than them with normal levels of HDL-c in adults, which indicated that HDL-c effectively modulate sICAM-1 in vivo [28], which was in line with the result on boys. It is well known that HDL-c had a protective effect to prevent the development of atherosclerosis by inhibiting the expression of adhesion molecules (ICAM-1, VCAM-1 and E-selectin) induced by cytokines on the cell surface and also acts as antioxidant and mitogen and also binds the lipopolysaccharide [35]. There is evidence that the incidence of cardiovascular disease (CVD) is greater in men, and it was suspected that sex hormones, particularly estrogen, have modulatory effects on the endothelium and circulating cells which have been implicated in vascular inflammation. Estrogens upregulate the synthesis, release and activity of protective agent on endothelial, and suppress the expression of pathogenic agents by endothelium via estrogen receptor (ER), Er*α* and Er*β*, on vasculature [36], while androgens are suspected to be proatherogenic [37]. Moreover, estrogen is able to reduce the fat accumulation in women [38] and showed that its level is higher than men/boys at any age [37]. The in vitro study exhibited that estrogen inhibits smooth muscles by activating potassium efflux and inhibiting calcium influx. Furthermore, estrogen inhibits vascular smooth muscle cell proliferation [39].

VCAM-1 levels in obesity with hypertension showed no significant difference with normotensive subjects on adults [40], or on healthy obese adults and unhealthy obese adults [32]. However, it showed lower levels on hypertensive boys with obesity, which contradicted with those results. The expression of VCAM-1 was inducted by proinflammatory cytokines such as IL-1 and TNF-*α* [35]. A study on adolescents showed that the increase of the BMI was associated with the elevation of hs-CRP and reduced HDL-c and indirectly elevated ICAM-1 but not VCAM-1 [27], which showed that inflammation is more profound influencing VCAM-1 expression. This finding is supported by the study on adults with left ventricular (LV) hypertrophy and sVCAM-1 was elevated [41, 42]. The increment of VCAM-1 levels depends on age, but there was no significant difference with gender on healthy adults [43]. It was reported that testosterone attenuated atherogenesis in vitro by converting testosterone into estradiol, which inhibits the expression of VCAM-1 (protein and mRNA) in dose-dependent manner by aromatase enzyme in human umbilical vein endothelial cells (HUVEC) [44], so we suspected the pubertal stage was influencing VCAM-1 levels in this study. However, we did not evaluate the pubertal stage of the subjects.

In adult population, ICAM-1, VCAM-1, and E-selectin were significantly correlated with fasting blood glucose (*r* = 0.59; *p* < 0.001); the increased ICAM-1 was suspected related to hyperglycaemia in type-2 diabetes mellitus subjects [45] which was in line with this study in both genders, but genetic study showed ICAM-1 was correlated positively with fasting insulin, HOMA IR, BMI, and waist circumference, and negative correlation with HDL-c [46]. Hyperglycemia decreases the expression of ICAM-1 and affects low proliferation of endothelial cells [47]. Endothelial cells exposed to high concentration of glucose leads to upregulation of the expression of adhesion molecules (ICAM-1, VCAM-1, and E-selectin) and proinflammatory cytokines due to the activation of poly ADP-ribose polymerase (PARP) enzyme. Moreover, glucose exposure also triggers the production of nitrotyrosine and induces the expression of adhesion molecules [48].

Based on gender, ICAM-1 was correlated with HDL-c in boys, although the correlation was very weak, which is in contrast with the studies conducted in paediatric population, ICAM-1 was correlated negatively with HDL-c [27, 49] and on adults [46]. Other studies on adults with hypercholesterolemia and ischemic heart disease showed that there was no correlation between ICAM-1 and HDL-c [50], which contradicted with this study. The in vitro study using human umbilical vein endothelial cells (HUVECs) treated with HDL-c from patients with T2DM showed that HDL-c stimulates the expression ICAM-1 and VCAM-1 on the cells' surface along with the protein kinase C (PKC) activity [51]. A study on T2DM patients showed that HDL-c from diabetic patients with CAD enhanced monocyte adhesion on the endothelial cells, which was correlated with HDL carbamyl-lysine (HDL-CBL). Culture study showed that carbamylated HDL promoted the adhesion of monocyte to endothelial cells, which promote induced upregulation of cell adhesion molecules' expression and activating NF-*κ*B/p65 signalling pathways. So, carbamylated HDL-c potentiated proatherogenic [52]. Moreover, dysfunctional HDL-c has proinflammatory effects on the endothelial cells [53], so we suspected that boys with obesity experience dysfunctional HDL-c, which induced the expression of ICAM-1. The elevation of 100 ng/ml ICAM-1 will increase the risk of cardiovascular mortality by 1.10-folds (95% CI (1.05–1.15)) and get worse due to the presence of hypertension and cardiovascular disease [54].

The in vivo study on mice showed that the increment of LDL-c levels increased the expression of ICAM-1 and VCAM-1 in aortic endothelial cells which were regulated by AP-1 and TNF-*α*. The expression pattern of ICAM-1 was pervasive, but VCAM-1 expression appears more focussed and less elevated [55]. This finding explained the weak correlation between LDL-c and ICAM-1. The atherogenesis process is initiated when the accumulation of LDL-c particles in the arterial wall is trapped in subendothelial space. LDL-c will undergo oxidation processes by oxidants such as nitric oxide and its products, and myeloperoxidases. One of the key events of atherogenesis is the accumulation of LDL particles in the arterial wall. Once entrapped in the subendothelial space, LDL undergoes modification through oxidation (oxidized LDL) by cell-derived oxidants, such as nitric oxide and its products, and myeloperoxidases, in which it induces the expression of adhesive molecules on endothelial cells [56].

Hypertension contributed to the initiation of atherosclerosis due to its association with inflammation [57] and sVCAM-1 [58]. Several studies indicated positive association between blood pressures with VCAM-1 in adults [59–61], which was in contrast with this study. However, gender influences the blood pressure regulation. Systole blood pressure 4 mmHg is higher in boys than girls aged 13 to 15 years , and 10 to 14 mmHg is higher in boys aged 16 to 18 years which showed that androgen, especially testosterone, plays a regulatory role on blood pressure, which made men at greater risk of cardiovascular and renal diseases than women at age-matched [62]. Adolescence is the time of growth spurt and the onset of puberty, characterized by hormonal changes [63] in which there is elevation on androgens, in boys, testosterone, which leads to the elevation of blood pressure [62]. This explains the negative relationship between blood pressure (systole and diastole) with VCAM-1; in other words, blood pressure in this study is likely linked with hormonal changes during pubertal period. This fact explains the significantly higher VCAM-1 in boys with MetS than girls with MetS, even girls with MetS had lower VCAM-1 levels than boys with non-MetS. An in vivo study showed that insulin concentration (10^−9^–10^−7^ mol/L) induced VCAM-1 expression and markedly increased TNF-*α* via NF-kB activation and IkB-*α* accumulation [64], so we suspect that hyperinsulinemia stimulates the expression of VCAM-1, not the blood pressure. Moreover, VCAM-1 was correlated with age and becomes an independent predictor of VCAM-1 levels, but ICAM-1 was not influenced by age and sex [50], which explained the levels of ICAM-1 which did not show a significant difference in this study.

Abdominal obesity was associated with lower adiponectin levels on children aged 9-10 years [65], and its levels were influenced by pubertal stage [66] and gender. The similar results were also seen in other studies [4], which support this finding. In healthy lean boy, adiponectin was reduced along with physical and pubertal development and was significantly lower than that in girls [67], which tend to be low at late obesity[68]. Waist circumference, for detecting central obesity, was correlated negatively with adiponectin levels in girls [4, 69], which was in line with this study; the levels of adiponectin was significantly lower in central obesity in girls. Based on gender, adiponectin is higher in girls than boys [67, 70] until adulthood [68] and thought to be influenced by sex hormones [71]. It was found that adiponectin was correlated with androgens, not testosterone and estradiol [68, 70, 71] but the results were still controversial. In girls, adiponectin thought to act as female fertility regulation [72], which was also supported by others, and adiponectin promotes gonadal activities [73]. That is why adiponectin affects ICAM-1 and VCAM-1 in girls in the path analysis. Adiponectin plays a role in steroidogenesis of ovarian. Together with insulin-like growth factor 1 (IGF-1), adiponectin improves the secretion of progesterone and estrogen in the animal model study [73]. A study conducted on postmenopausal women concludes that more androgenic sex hormone profiles (high testosterone) were associated with higher flow-mediated dilatation (FMD), which may impair the vasculature [74]. Endogenous estrogen affects endothelial cells by upregulating the NO synthase, protects from lipid metabolism, and reduces oxidative stress [75].

A study conducted on adults showed that plasma P-selectin correlated positively with triglycerides only in the overweight/obese group [76]. The in vivo study on mice fed with high fat diet showed a significant correlation between sICAM-1 with body weight and visceral fat or central obesity due to the expression ICAM-1 mRNA on the adipose tissue, which is higher on male mice than female mice, followed by the increment of IL-6 and monocyte chemoattractant protein-1 (MCP-1) as the consequences of atherogenic lipid (LDL-c, cholesterol, and triglyceride) in the diet [77]. But the study to assess the direct effect of triglycerides on ICAM-1 is limited. However, this study found out whether girls or boys with obesity experience the elevation of ICAM-1 due to the elevation of triglyceride, although it did not have a direct or indirect effect on VCAM-1, which was supported by ex vivo study using postprandial triglyceride-rich lipoproteins (PP-TGRLs) combined with TNF-*α* stimulation (at dose 0.3 ng/mL), increasing the expression of VCAM-1, ICAM-1, and E-selectin by 10–15%. The transcription and expression of VCAM-1 depends on postprandial serum triglycerides (PP-sTG) and waist circumference [78].

In girls, adiponectin had a negative influence on ICAM-1, which was in line with other studies [79], in which adiponectin acts to suppress the expression of ICAM-1. In this function, adiponectin acted as protective agents against various obesity-linked diseases, including cardiovascular disorders, by attenuating inflammatory response via signalling pathways in various cell types [80]. The finding was in line with this study. The activity was mediated via its receptors, adiponectin receptor 1 (AdipoR1), expressed abundantly in the skeletal muscle, and adiponectin receptor 2 (AdipoR2) found predominantly in the liver. Both receptors are transmembrane G-protein-coupled receptors and found on macrophages, endothelial cells, and smooth muscle cells within atherosclerotic plaques. These receptors were able to activate AMP-activated protein kinase (AMPK), p38 mitogen-activated protein kinase (p38 MAPK), and peroxisome-proliferator-activated receptor-*α* (PPAR-*α*) and to stimulate fatty acid oxidation and glucose uptake [5]. This functional role of adiponectin was exerted in dose-dependent manner (5 to 25 *µ*g/mL), in which TNF*α*-induced adhesion expression, including ICAM-1 and VCAM-1 on the in vivo study, was then inhibited [6].

## 5. Conclusion

VCAM-1 was significantly higher on boys which showed that boys had higher risk of atherosclerosis than girls, while ICAM-1 showed no significant difference on both gender and metabolic states. Adiponectin showed a protective effect by lowering ICAM-1 and VCAM-1 directly on girls.

## Figures and Tables

**Table 1:** Subject's characteristics.

Characteristics	Non-MetS (*n* = 83) (mean ± SD)		MetS (*n* = 42) (mean ± SD)		*p* value
	Boys = 51	Girls = 32	Boys = 37	Girls = 5	
Age (months)	180.76 ± 13.35	182.44 ± 18.58	180.03 ± 17.07	184.60 ± 24.42	0.891^a^
Body weight (kg)	84.82 ± 13.51	78.30 ± 9.44	92.64 ± 14.43	86.86 ± 8.11	<0.001^b^
Body height (cm)	162.76 ± 7.38	156.05 ± 5.71	167.38 ± 8.56	163.40 ± 5.07	<0.001^a^
BMI	31.80 ± 4.48	32.18 ± 3.97	32.94 ± 3.99	32.75 ± 5.15	<0.001^a^
Waist circumference (cm)	99.92 ± 9.17	90.13 ± 8.73	103.33 ± 7.91	91.50 ± 5.15	<0.001^a^
Hip circumference (cm)	108.28 ± 8.56	108.53 ± 7.82	110.48 ± 8.64	113.20 ± 6.53	0.419^a^
Systolic BP (mmHg)	120.39 ± 13.55	118.75 ± 9.07	130.54 ± 10.46	132.00 ± 8.37	<0.001^c^
Diastolic BP (mmHg)	77.55 ± 8.74	77.18 ± 6.83	86.35 ± 10.32	86.00 ± 8.94	<0.001^c^
Total cholesterol (mg/dL)	163.41 ± 36.02	175.06 ± 33.33	174.10 ± 25.20	192.80 ± 18.21	0.115^a^
LDL-c (mg/dL)	104.12 ± 29.63	113.50 ± 26.97	116.03 ± 22.88	128.20 ± 18.09	0.077^a^
HDL-c (mg/dL)	44.47 ± 6.29	46.03 ± 7.62	37.30 ± 5.41	40.80 ± 3.70	<0.001^a^
Triglycerides (mg/dL)	102.72 ± 62.60	101.81 ± 63.24	148.10 ± 68.57	149.60 ± 36.39	0.001^c^
Fasting blood glucose (mg/dL)	86.27 ± 6.25	85.19 ± 4.01	86.37 ± 5.76	88.80 ± 7.26	0.591^c^
Fasting blood insulin (mU/L)	18.74 ± 7.57	21.13 ± 11.45	28.91 ± 18.53	24.43 ± 9.54	0.021^c^
HOMA IR	3.94 ± 1.58	4.45 ± 2.35	6.20 ± 4.02	5.38 ± 2.17	0.019
Adiponectin (ng/ml)	15.80 ± 7.55	16.42 ± 78.81	11.86 ± 4.08	11.93 ± 7.54	0.068^c^
ICAM-1 (pg/ml)	938.89 ± 1,568.767	899.59 ± 1,546.17	3,274.02 ± 15,207.28	554.46 ± 254.83	0.066^c^
VCAM-1 (pg/ml)	13.41 ± 13.04	5.28 ± 6.99	15.57 ± 22.24	4.52 ± 1.45	<0.001^c^

^a^One-way ANOVA, post hoc Bonferroni; ^b^One-way ANOVA, post hoc Tamhane; ^c^Kruskal–Wallis, post hoc Mann–Whitney, significant if *p* < 0.05. BMI: body mass index; BP: blood pressure; LDL-c: low density lipoprotein cholesterol; HDL-c: high density lipoprotein cholesterol; HOMA IR: homeostasis model assessment of insulin resistance; ICAM-1: intercellular adhesion molecule; VCAM-1: vascular cell adhesion molecule 1.

**Table 2:** The levels of adiponectin, ICAM-1, and VCAM-1 on MetS risk factor on boy adolescents with obesity.

MetS risk factor	Adiponectin, ng/ml (mean ± SD)	ICAM-1, pg/ml (mean ± SD)	VCAM-1, pg/ml (mean ± SD)
*Gender: boys (n* *=* *88)*			
Central obesity			
Yes (*n* = 84)	13.95 ± 6.49	1,910.21 ± 10,145.93	14.06 ± 17.12
No (*n* = 4)	18.36 ± 8.43	2,141.19 ± 2,656.01	19.72 ± 25.49
*P* value	0.203^b^	0.072^b^	0.650^b^
Hypertension			
Yes (*n* = 37)	13.10 ± 4.10	520.94 ± 353.18	9.01 ± 4.98
No (*n* = 51)	14.90 ± 7.88	2,936.22 ± 12,989.08	18.17 ± 21.78
*P* value	0.651^b^	0.571^b^	0.001^b^
Hyperglycaemia			
Yes (*n* = 2)	22.65 ± 16.31	812.76 ± 50.84	12.54 ± 1.48
No (*n* = 86)	13.92 ± 6.29	1,946.47 ± 10,036.91	14.36 ± 17.62
*P* value	0.356^b^	0.370^b^	0.484^b^
Hypertriglyceridemia			
Yes (*n* = 38)	12.55 ± 4.89	3,391.82 ± 14,980.53	16.33 ± 21.84
No (*n* = 50)	15.36 ± 7.46	802.65 ± 1,546.84	12.79 ± 13.17
*P* value	0.070^b^	0.613^b^	0.393^b^
Low level of HDL-c			
Yes (*n* = 38)	12.49 ± 5.26	3,138.68 ± 15,018.66	15.72 ± 21.81
No (*n* = 50)	15.40 ± 7.25	995.04 ± 1,567.86	13.26 ± 13.30
*P* value	0.114^b^	0.004^b^	0.800^b^

^b^Mann–Whitney *U*, significant if *p* < 0.05. HDL-c: high density lipoprotein cholesterol; ICAM-1: intercellular adhesion molecule; VCAM-1: vascular cell adhesion molecule 1.

**Table 3:** The levels of adiponectin, ICAM-1, and VCAM-1 on MetS risk factors on girl obese adolescents.

MetS risk factor	Adiponectin, ng/ml (mean ± SD)	ICAM-1, ng/ml (mean ± SD)	VCAM-1, ng/ml (mean ± SD)
*Gender: girls (n* *=* *37)*			
Central obesity			
Yes (*n* = 30)	14.25 ± 6.09	652.54 ± 299.46	4.23 ± 1.25
No (*n* = 7)	22.53 ± 13.36	1,711.79 ± 3,314.50	9.24 ± 14.92
*P* value	0.016^a^	0.614^b^	0.985^b^
Hypertension			
Yes (*n* = 5)	17.45 ± 7.65	511.24 ± 370.25	5.70 ± 2.50
No (*n* = 32)	15.56 ± 8.59	906.34 ± 1,541.48	5.09 ± 6.95
*P* value	0.646^a^	0.339^b^	0.213^b^
Hyperglycaemia			
Yes (*n* = 1)	9.08 ± 0	692.88 ± 0	3.82 ± 0
No (*n* = 36)	16.00 ± 8.44	857.39 ± 1,462.46	5.22 ± 6.59
*P* value	0.424^a^	0.925^b^	0.888^b^
Hypertriglyceridemia			
Yes (*n* = 13)	13.93 ± 9.91	1,388.90 ± 2,355.17	7.07 ± 10.86
No (*n* = 24)	16.83 ± 7.45	562.63 ± 333.98	4.15 ± 1.26
*P* value	0.042^a^	0.101^b^	0.474^b^
Low level of HDL-c			
Yes (*n* = 12)	17.56 ± 9.20	1,293.63 ± 2,504.92	7.34 ± 11.29
No (*n* = 25)	14.98 ± 8.05	641.42 ± 317.18	4.14 ± 1.28
*P* value	0.390^a^	0.922^b^	0.897^b^

^a^Independent sample *T*-test; ^b^Mann–Whitney *U*, significant if *p* < 0.05; BP: blood pressure; LDL-c: low density lipoprotein cholesterol; HDL-c: high density lipoprotein cholesterol; HOMA IR: homeostasis model assessment of insulin resistance; ICAM-1: intercellular adhesion molecule; VCAM-1: vascular cell adhesion molecule 1.

**Table 4:** The correlation between adiponectin, ICAM-1, VCAM-1, and the MetS parameters (blood pressure, lipid profile, and fasting (blood glucose) based on gender.

Physical measurements and MetS parameters	Adiponectin		ICAM-1		VCAM-1	
	*r*	*p*	*r*	*p*	*r*	*p*
*Boys (n* = *82)*						
Waist circumference	−0.126	0.242	−0.114	0.290	−0.031	0.776
Hip circumference	−0.127	0.237	−0.076	0.483	0.005	0.964
BMI	−0.065	0.549	0.047	0.665	0.040	0.710
Fasting blood glucose	−0.087	0.418	0.217	0.043	0.002	0.986
Fasting insulin	−0.272	0.011	−0.011	0.918	−0.009	0.932
Triglycerides	−0.174	0.105	0.145	0.177	0.130	0.229
Total cholesterol	0.035	0.744	0.145	0.177	0.165	0.123
HDL-c	0.155	0.148	0.261	0.014	0.032	0.767
LDL-c	0.005	0.964	0.118	0.274	0.228	0.033
Systole-BP	−0.051	0.634	0.047	0.666	−0.265	0.013
Diastole-BP	−0.049	0.654	−0.144	0.180	−0.264	0.013

*Girls (n* = *38)*						
Waist circumference	0.070	0.681	−0.013	0.941	0.054	0.752
Hip circumference	−0.056	0.743	0.021	0.902	−0.035	0.835
BMI	0.154	0.362	−0.204	0.226	−0.160	0.345
Fasting blood glucose	0.072	0.670	0.422	0.009	0.111	0.513
Fasting insulin	−0.224	0.182	0.183	0.279	0.080	0.638
Total cholesterol	0.044	0.797	−0.005	0.977	−0.100	0.557
Triglycerides	−0.199	0.239	0.138	0.416	0.271	0.104
HDL-c	0.163	0.336	0.106	0.533	−0.035	0.839
LDL-c	0.119	0.484	−0.147	0.385	0.224	0.182
Systole-BP	0.043	0.801	−0.072	0.671	−0.076	0.654
Diastole-BP	−0.046	−0.785	−0.077	0.649	0.116	0.493

^*∗*^Spearman correlation, significant if *p* < 0.05.

## Data Availability

All data used to support the findings of this study are included within the article.
